# Heterodox modeling: practicing well-tuned provisioning or commoning with networked multi-agent environments

**DOI:** 10.1007/s43253-023-00109-7

**Published:** 2023-10-24

**Authors:** Shintaro Miyazaki

**Affiliations:** Humboldt-Universität zu Berlin, Berlin, Germany

**Keywords:** Commoning, Heterodox Modeling, Gaming, Provisioning, Lifenet, B15, B25, B51, C63, P41, Z13

## Abstract

Market-driven, profit-oriented, mainstream neoclassical economics is increasingly being challenged by alternative approaches such as heterodox economics. This article contributes to broader discussions in this field, especially of social provisioning, and suggests that integrating perspectives from the overlapping fields of media history and history of economy could not only provide valuable insights and attract more supporters, but even initiate a bottom-up transformation process. Historical knowledge regarding how neoclassical economics gained mainstream status in the early postwar decades provides hints on how to popularize non-profit-driven, well-designed approaches to social provisioning, often referred to as commoning. More specifically, the article firstly addresses the necessity of a large-scale appropriation of computational methods, procedures, tools, media, and models to experiment with economic issues, which are usually applied mostly by mainstream profit-driven approaches. Secondly, it presents the implications of such a practice, which I tentatively refer to as heterodox modeling, while remembering the 1960s North American context of business simulation games and their role within the large-scale effort to educate and train the newly-defined class of “business managers.” The article, thirdly, theorizes heterodox modeling as being based on a still imaginary, not-yet implemented, networked multi-agent online environment, which would integrate the modular programming of agent-based models, group exercises, role-playing, gaming, and testing of operations and processes within large-scale socio-ecological networks of commoning. Finally, the article argues not only to model heterodox economic theories, but at the same time to model in heterodox ways and highlight associated implications.

## Introduction and background

Heterodox economics provides alternatives to mainstream neoclassical economics, which emerged in the late nineteenth century to define economy exclusively as market exchanges. According to economists Tae-Hee Jo and Zdravka Todorova, this dissenting field includes broader societal and historical accounts of provisioning and inquires how economies get organized, maintained, and reproduced. Provisioning, a key concept in some threads of heterodox economics, is meant as the activity of preparation, supply, and maintenance, more generally as the activity of sustaining life itself. Etymologically, the term provision is related to foresight, foreseeing, or looking ahead, as well as to prediction. Therefore provision also implies models of those things which are about to happen. Arguing in an escalated way, heterodox economics is consequently tightly coupled with other ways of modeling economic activities. It is furthermore “situated in the long intellectual tradition, which concerns the material basis of the society as an outcome of the open-ended interaction or struggle between human beings and nature, between social classes, and between agency and social structures” (Jo and Todorova 2017: 35). This framing of heterodox economics comes quite close to what is sometimes called evolutionary political economy, since it inquires into the long evolutionary history of human society and organic and material environments in order to provide new insights for an accurate critique of present and past political economies.

This article argues that perspectives from economic history, media history, and the history of software focusing on computing, simulation, and modeling might – in concert with heterodox economics – provide useful insights not only to get broader support for the latter field and to accumulate a critical mass of supporters and prospective practitioners, but also to initiate a bottom-up transformation process. The argumentation concentrates on simulation and its role in envisioning post-capitalist utopias of provisioning with the help of networked, cooperating users and agent-based modeling. It proposes a historically-informed practice, tentatively called heterodox modeling, as a method to critique, construct, conceptualize, experience, and practice an activity called commoning. Commoning refers to commons-based and solidarity-oriented activities of living together, of production and reproduction, of well-tuned provisioning, if you will. Heterodox modeling, furthermore, subsumes two meanings: Firstly, it proposes modeling heterodox economic theories. But secondly, at the same time it also proposes modeling in heterodox ways.

The article begins by situating the success of mainstream neoclassical economics as a result of a Cold-War effort since the 1950s and describes the role of business schools and the emergence of simulation and gaming in economics influenced by experiences and knowledge accumulated during WWII. In a next step the article argues for heterodox methods of economic modeling and describes some key aspects of heterodox modeling: firstly with a historical contextualization of bottom-up agent-based modeling, which emerged during the 1990s; and then secondly along two specific contexts entangled with crucial aspects of heterodox modeling. Instead of a conclusion, the article will finally articulate what it means not only to model heterodox theories, but model in heterodox ways by referring to a wider set of playful, but critical, experimental and participatory practices. In order to envision post-capitalist utopias, I argue, we need to change not only “the what,” but also “the how” of modeling and simulation.

## Training

For a rough genealogy of the success of mainstream neoclassical economics and using the rise of neoliberalism since the late-1970s Cold War as a context, the Sputnik crisis of late 1957 is especially worthy of focus. The Soviet Union’s successful launch of the first man-made satellite to go into orbit of the Earth led not only to the passing of the US National Defense Education Act (NDEA) in 1958, but also accelerated further investment by the ruling US bourgeois class into the training of economists and managers to win the “battle with communism,” as suggested by a history of US management written by leadership scholar and Dean of Harvard College Rakesh Khurana (see 2007: 239–240). According to Khurana, large capital from funds such as the Ford Foundation and the Carnegie Corporation of New York flows into universities and new business schools, and this has greatly affected faculty composition, research, and curricula (2007: 236, 250), leading to an accelerated success not only of mainstream neoclassical economics, but also of quantitative tools taken over from WWII.

Military leaders, managers, and organizational experts had created an arsenal of quantitative tools such as linear programming, systems analysis, computer simulations, network analysis, queuing theory, and cost accounting systems to control and administer the war machine. [...] The importation of these technical innovations into business gave rise to a different conception of what it meant to be a professional manager. (2007: 203)

Among those to profit from such funding for optimized management based on new technologies was the Carnegie Mellon School of Industrial Administration^1^ led by George Leland Bach from the late 1940s on, and with faculty members such as Herbert Simon (since 1949), the latter going on to receive the Nobel Prize in Economics in 1978. Another lesser-known faculty member from a much younger generation than these two economists was Kalman J. Cohen, who was a key figure for the field of management simulation games or business simulation games.

The first business simulation game was the *Top Management Decision Simulation*, an early business simulation game commissioned by the American Management Association in collaboration with the RAND Corporation, the Naval War College, and the International Business Machines Corporation (IBM) and which was documented in a large volume published in 1957. The list of institutions involved clearly reveals the links between military and industry, which also crystallized itself into the fact that business games in that publication were described as having been derived from wargames.^2^ Many variations, adaptions, and extensions followed within a few years and business simulation games have been massively instrumentalized for many years in management education, economics, and research on markets, logistics, and organization. Rolf F. Nohr, a German media studies scholar, most recently situated these games as advanced educational tools, firstly, incorporating a “specific rationality in terms of managing the future” (2023: 76) and secondly, “by means of which middle and upper management were to train and improve their decision-making skills” (77). Mary Morgan, historian of economics and more broadly concerned with the role of models and simulation in 20^th^-century economics drew heavily on work by Martin Shubik, who in 1960 not only compiled an extensive “Bibliography on Simulation, Gaming, Artificial Intelligence and Allied Topics,” but moreover described the new digital computer as “laboratory equipment for economics” and thus changing its methods in ways similarly profound to how the adoption of “the microscope for biologists” had done (Shubik 1960: 908; Morgan 2012: 320–321). Shubik also theorized business gaming as a man–machine simulation emphasizing the interplay between simulation as a rather machine-based process, and role-playing as a human-based mode of interaction. Around the same time period, Kalman J. Cohen and Richard M. Cyert argued the same, but highlighted the dynamism computer-based economic simulations offer: “they provide a language within which complex dynamic models can be constructed” (Cohen and Cyert 1961: 127). Simulations and models both allow dynamic theories and programs, but also make programming and theorization more dynamic.

To summarize, four aspects seem notable: Firstly, mainstream modeling, simulation, and gaming comprise crucial ingredients for the success of mainstream neoclassical economics. Secondly, these rather new computational management tools stemmed to a large degree from the war machinery of WWII. Business simulation games are embedded heavily in military-industrial networks. Thirdly, these tools therefore inherit substantially aggressive or passive-aggressive strategies of competition, identity-conservation, prediction, extraction, exploitation, exclusion, and discrimination for the sake of sales, revenue, and profit.^3^ And fourthly, modeling, simulation, and gaming can massively accelerate the education of new communities and groups of people sharing the same principles, assumptions, biases, ideologies, and practices. Consequently, I argue for heterodox modeling, simulation, and gaming as methods of heterodox economics and evolutionary political economy, which simply and truly counter the second and third of the aforementioned aspects. Heterodox modeling should ideally operate in favor of well-tuned planet-wide social provisioning, thus of commoning, and furthermore take quite seriously the historical contingency of mainstream neoclassical economics and the strategy of dissent embedded in the meaning of “heterodox,” and explore the implications of that contingency up to the point where the concepts of economics and of modeling will almost dissolve.

## Open-ended inquiry

At least two different levels of scales, which are themselves recursively applicable to different aspects and fields of critique and analysis, become pertinent.^4^ There is the slightly higher or larger scale of rather complex models of commoning, while the lower or smaller scale is about models, which due to their simplicity and bottom-up perspective offer explainability of effects in the realm of everyday interactivity. Before concretizing how these scales could potentially complement each other, the following section will first situate the epistemological shift from a larger scale to a lower scale.

The methods of computer-based modeling, simulation, and gaming changed drastically in the late 1980s due to new ways to plan and program software introduced with the popularization of object-oriented programming and the continued reduction of the cost and size of computer hardware. The change from so-called structured programming to object-oriented programming has been pertinently described by the German computer scientist and software historian Jörg Pflüger. While up to the end of the 1960s algorithms and software had been written by experts, the increasing commercialization of software production led to the industrialization of programming. Emerging around 1980, at the latest, object-oriented programming went along with attempts to decentralize, to de-hierarchize, and to modularize the programming work again. Along with component-oriented bottom-up processing, new sorts of requirements came up, which became tangible primarily in the emerging field of computer-based simulation and modeling, requiring the deconstruction of the tree-like block structure of older programming languages such as ALGOL into networked, operational units, later called objects, that are active or in a waiting state and can interact with each other (Pflüger 2004: 297). With the dissemination of object-oriented programming environments such as Smalltalk, complex, decentralized networks of algorithms triggering the operations of individual agents or objects can be programmed, wherein a meta framework regulates when, how, or under which conditions a certain object is to be called and later deleted. Thus software can firstly be designed, tried out, tested, and varied more easily, which secondly created optimum conditions for new bottom-up, object-level, and agent-based modeling methods leading to simulations of swarm behavior in shoals of fish or flocks of birds, explanations of traffic jams, or tipping points in segregation behavior in urban settings.^5^

While the previous approaches to modeling were rather based on aggregate effects and overall processes usually modeled after differential equations or feedback systems as in system dynamics, agent-based modeling shifted the focus from modeling-aggregated effects on a higher level to the low level of those sub-aggregate processes and bottom-up interactions amounting to some overall behavior. This introduction of the bottom-up level of individually-programmed agents enabled new approaches related to the emergence of complexity science in the 1990s. In parallel, such low-level perspectives – for example on agent-level social interactions and rules – also made it possible to integrate game-theory-inspired decision-making models into larger models. Andy Clark, Fellow of the British Academy and neurophilosopher, with reference to the 1990s argued that our biological brains, in concert with new computational and networked media, could possibly grow into hybrid minds better able to understand the kinds of systems in which they themselves participate. A concrete example of such a hybrid network of brain, body, and new technologies is, according to Clark, the StarLogo programming language and environment developed by the MIT Media Lab and Mitchel Resnick in the 1990s (Clark 2003: 159). Whereas Logo, StarLogo’s predecessor project directed by Seymour Papert, could merely be used to program one single drawing pen (or Turtle), StarLogo's rigorous application of object-oriented programming made it possible to have several thousand agents interact with each other as software objects in an artificial architecture. StarLogo can be used to model behavior including the foraging behavior of ants, the formation of traffic jams, the spread of forest fires, or even the dynamic configuration of swarms (Resnick 1994: 49–117).^6^ The idea of experiencing, testing, and rehearsing agent-based models in a bodily and situated way, through role-playing and group exercises similar to business games in order to better understand them, probably emerged early on during the development of StarLogo. This was tested at conferences, as described by Resnick and Uri Wilensky. In a playful gesture, the group exercises were called StarPeople. StarLogo and StarPeople form a hybrid brain-body-media network. Here, the primary goal was to understand and experience the unfolding and behavior of complex systems through interactive movement games. This constellation comes very close to the idea of man–machine simulation as articulated by Shubik three decades earlier.

While the old business simulation games of the 1960s rather did not model processes on the low level of bottom-up interactions and while they operated from a top-down perspective, the new agent-based models would allow a combination of bottom-up and top-down perspectives in theory. The few descriptions of such exercises, conducted in the 1990s at conferences on learning and education and in classrooms, showed that they experimented more with the directly embodied modeling and did not design some man–machine interactions in a literal way, but the agent-based models enacted by humans were most meaningful when compared with computer-simulated agent-based models. The simplest and often the first exercise for an ad hoc brain-body-media network was one about decentralized synchronization through hand clapping (Resnick and Wilensky 1998: 157). The next exercise was one about decentralized communication, that is, the decentralized and self-organized formation of groups in networks. To do this, the group was divided into six unevenly distributed subgroups. Each participant had to keep their assigned group – indicated by receiving a piece of paper with a number between one and six – to themselves. The goal of each round of the exercise was to find more group members. In between, their experiences were shared and discussed. The first round started without any restrictions. The groups quickly formed, some loudly announcing their group number. In the second round, a ban on speaking was introduced. Participants began to show each other the pieces of paper, showing cohesion and moving together. In the third round, everyone was blindfolded, and at the same time whispering was allowed. Now it took a long time for the groups to form; often individual participants were “left over” or lost. Some developed search strategies by holding hands, for example, forming an elongated structure that allowed them to search the room more quickly. According to Resnick and Wilensky, the three exercise rounds in which different situations of communication conditions were tested and practiced offered reflections on the different roles of centralized or decentralized local structures, of chance, probability, of sensorial or physical conditions, and the role of effectiveness or even feasibility of actions in certain constellations (161).

The most important learning the human-based performance and gaming of agent-based models might provide is the relation between bottom-up operativity of individual agents and sometimes aggregated effects which overall remained unseen due to tipping points and non-linear mechanisms emerging from seemingly innocuous individual actions. Often there is no direct causality that could be understood by either linear or intuitive means. This knowledge, and the experience gained after practicing these exercises,^7^ becomes critical and decisive, especially when it comes to understanding complex systems and how bottom-up processes lead to aggregated effects in unexpected, non-linear manners. The criticality of such a perspective has also been considered in heterodox economics, most prominently by German-speaking scholars such as Hardy Hanappi and Manuel Scholz-Wäckerle (see 2021), but while there are numerous interesting models in their principal spirit, models specifically addressing issues of well-tuned social provisioning, or commoning, as a means of providing real alternatives to market-based and exchange-value-oriented production economies, are still rare or currently in the making (Gerdes et al 2023). It seems that even heterodox economists with expertise in agent-based modeling are mostly occupied with modeling existing economic situations and issues (see Elsner et al 2015 or Cogliano and Jiang 2016), while others such as David Laibman have a rather rough understanding of what a model is, although his proposal for a synthesis of different models within the framework of centralized versus decentralized planning and quantitative versus qualitative rules is surely pertinent to computer-based heterodox modeling (see 2022).^8^ Numerous agent-based models of commons-based productivity, urban commons and commoning exist (see Feinberg et al 2023), but many do not propose real alternatives but instead shift perspectives as for instance with the needs and limits framework, which often still relies on concepts such as wage labor and income (Foramitti 2023). Many agent-based models of processes related to commoning, furthermore, do not use the term, but are conceptualized by the Social-Ecological Systems framework (Lippe et al 2019; Schlüter et al 2019). Others like myself with limited training in modeling and situated in context of artistic and experimental design research collaboratively conducted tentative modeling experiments with confined outreach (Savic et al 2020). Synthesizing and organizing all these different approaches into an interlinked and well-connected field is still due. This article aims to provide some more approaches for new beginnings.

Two rather disparate models offering complementary views will finally provide useful entry points for an ongoing open-ended inquiry about the agents, processes, rules, and environments which could make up a working model of commoning and well-tuned provisioning. The first model (A) is a rather classic one formulated around 1990, not in the context of complexity science, but instead by Elinor Ostrom drawing heavily on game-theoretical arguments (see 1990). The second model (B) has been described more recently by Duncan K. Foley and is called Lifenet (see 2020). Ostrom’s work on common-pool resources (A) is widely regarded as providing the fundamentals of commoning and provides the basis for theorizing heterodox approaches going beyond purely profit- and market-driven modes of social provisioning, and describes more resource-conserving and eco-friendly production methods. Referring to a simple game she calls the “Hardin herder game” with sheep, grassland, and herders, which is based on a game theory classic called the prisoner’s dilemma, Ostrom argues for the importance of communication between all stakeholders. The game is for two stakeholders, with Alpha and Beta as herders, and is also called a zero-sum game. Without any communication between Alpha and Beta nor any central instance operating with sanctions, there will always be an incentive not to cooperate or share in solidarity, since the non-cooperative, selfish winner-takes-all strategy potentially results in more profit. But in the case that both Alpha and Beta choose this promising strategy, they will soon deplete the resources and lose everything. If there is a central agency with fully reliable information and with permission to order sanctions against cases of non-cooperation, there is no incentive *not* to cooperate; instead, Alpha and Beta start to cooperate. Even in the case that there is such an agency, which is only reliable up to 75%, the cooperative strategy still promises more sustainable profit. There is a high cost for everybody involved to maintain such a central agency. Hayekian ideologists would lament that only one individual agency can never fully know what is happening and rationality is always bounded. Ostrom offers here an alternative approach: i.e., when Alpha and Beta communicate with each other and start to self-organize, monitor each other, complete missing bits of information, and formulate rules and sanctions. Compared with the central agency, which induces additional costs, in this last variant these costs would also be distributed equally between Alpha and Beta.^9^ This alternative proposes changes in the structure of zero-sum games, which are usually rather classic, but Ostrom’s model is surely slightly heterodox, when she argues for “simple mechanisms that illustrate alternatives to those that normally are presented as the dominant solutions” (Ostrom 1990: 18). It would surely be worth turning this game-theoretical model into a playable game similar to the mentioned business simulation games. Ostrom’s work did not halt in the 1990s. More than a decade later, Ostrom adds agent-based modeling to her toolkit and proposes, in collaboration with Marco A. Janssen, a simulation model of common-pool resource provisioning. This would have a focus on the conditions during which users cooperatively agree on a known rule about how to best consume the resource, establishing this as counter to the unorganized liberalist wild-west strategies which lead to resource depletion (2007: 68–69). The model is quite complicated with many parameters and will surely inspire further detailed studies and re-experimentations of it which are yet to be conducted. Models in the vein of Ostrom and Janssen’s agent-based model provide crucial ideas for the heterodox modeling of well-tuned provisioning and commoning regarding, for example, the very seminal question on the importance of a self-organized, decentralized participative design of rules, of protocols, and of insights regarding crucial and critical factors.

The Lifenet model as formulated by Duncan K. Foley (B) provides an example for a slightly higher-level perspective. Still, Foley argues in the spirit of bottom-up thinking, and refers to prestigious research institutions in complexity science such as the Santa Fe Institute and for a socialism informed by complexity science, if you will. In this article here, such a perspective is also called well-tuned provisioning or commoning. Foley firstly proposes in a very Marxist way that commodity exchange is to be avoided, since.

even if it were possible hypothetically to equalize ownership of productive resources completely at one moment of time, there are powerful equilibrium tendencies of commodity exchange that would tend to reproduce a highly unequal distribution of income and wealth. (Foley 2020: 320)

His model also assumes that the elimination of private property in the means of production needs to get combined with an equal distribution of ownership of these means of production. This means not only turning resources into commons, but even the means and techniques of production. A commons includes not only material resources, but at the same time the technical and logistical means to produce them, including less energy-intensive, quasi-symbolic resources and forms of knowing embedded in both organic bodies and non-organic structures, machines, institutions, networks, and feedback control systems. Foley proposes an alternative set of social relations of social provisioning he calls “Lifenet,” which is based on a network of peer production initiatives^10^ which distribute their outputs freely to all participants, while all “agree not to re-sell” the output “as commodities for money.” Thereby all participants own a “Lifenet account,” which records individual contributions and withdrawals of Lifenet products in a central database (324). In Foley’s model such an account allows planning and monitoring. The open question is how to find a mechanism and signaling system, which “can adjust shortages and surpluses of particular products” (325). This would need specification and experimentation in further work.

What makes Foley pertinent for this article’s argumentation is that he formulates the idea of an “interactive computer game” (ibid.), which might help with realizing such a system. He remarks that “[o]ne advantage of the Lifenet fantasy is that it represents the transition to socialism as a cumulative process of day-to-day choice” (328). This highly constructive approach quite directly connects Lifenet with the aforementioned context of business simulation games. When Foley proposes to form a network of peer production initiatives, he assumes that they cooperatively share, organize, and manage resources and work as commons. Ostrom’s perspective and models inquired how and under what conditions forms of cooperation get accepted and negotiated among peer production operators, workers, and users who find themselves within the same socio-ecological situation, framework, and system. Here, both models partly diverge, since Foley proposes a real-life perspective on social provisioning assuming, in societies of late capitalism, that manifold networks, components, and processes work together, while Ostrom at least in her work from the 1980s and 1990s seems to argue more abstractly or refers to alternative real-life socio-ecological systems in non-industrialized regions either as historical cases or as anthropologically researched fields. Foley’s model is attractive for heterodox modeling, since it offers a polystructural, modular perspective, but is at the same time highly concrete and applicable to everyday life. Ostrom’s insights and models could operate as kernels of Lifenet’s productivity, but how products and consumers of several peer production networks find each other, and how this provisioning gets finely tuned in order that it works, is still an open question. Foley’s proposal to program a game-like environment with playful and virtual elements combined with computational and calculative procedures would help users in their effort for communal and solidarity-based resource sharing, production, and consumption, but, as I argue, it might be even more powerful and unleash its potential by going one step further. I propose to theorize and plan a large-scale effort, similar to the professionalization of business schools back in the 1960s, which includes collective practices of designing a programmable environment. This would imply a kind of massively-used programming environment for agent-based models playable by several interconnected users and with their source code being accessible for collective sharing, improvement, and critique. It would be an environment for heterodox modeling, which makes commoning more accessible, and therefore commonizes commoning and provisions provisioning, if you will.

## Rehearsing

Instead of a conclusion, the article articulates in this last section what it might mean not only to model heterodox theories, but to model in heterodox ways. Applied to what has been developed so far, this implies three tentatively-derived aspects and one last proposal.

Firstly, modeling and gaming as concepts need serious reconsideration,^11^ especially in terms of the assumed differences between reality, model (simulation), and game. A model is not a representation of reality; there is actually no such full representation thereof. For media studies scholar Claus Pias, simulations – and models, if you will – are “always furnished with a hypothetical index” and they generate “instead of certainty […] an uncircumventable spectrum of opinions and interpretations” (2011: 52). While models are, in the realms of profit-driven application, instrumentalized to reduce uncertainty, models elsewhere actually can – and in the realm of science often do – increase uncertainty. Gaming likewise seems to be unserious, but who really has the authority to judge what is serious or not. Gaming, playing, and pretending can all become activities with planetary impacts. As financial market crashes continuously show, in capitalism the realm of the symbolic-abstract, seemingly immaterial can have a serious effect on society. To overcome this dichotomy of gaming and real life, I propose to consider rehearsing, exercising, and practicing as terms and concepts enriching the meaning of modeling and gaming. Furthermore, heterodox variants of gaming have been explored for over a decade. In the early 2010s, Mary Flanagan, a game designer and scholar, proposed that

a hypothesis for activist gaming is that a well-crafted approach to embedding certain ideologies (interventionist strategies) in design will have the capacity to alter the practices on both the part of conscientious designers and artists as well as the players. (Flanagan 2013, 15)

With reference to the early-20^th^-century artistic practices of Dada she conceptualizes gaming and play as practices of research, which offer reflection and critique of everyday concerns. And she argues for ways to design gaming “to unplay, reskin, rewrite, and, in some cases, actively redefine culture” (2013, 139).

Secondly, I propose to reconsider modeling and gaming as practices solely reserved for experts in academic settings comprising rather small groups certainly linked to a community, but this communal aspect deserves more attention and engagement. Toxic, authoritarian, racist, and misogynistic networks of mostly male extreme right-wing gamers for example misuse Roblox, a commercial online game platform and game creation system allowing users to program games and play games created by other users. Heterodox modeling needs similar platforms to popularize ideas and principles such as commons-based peer production, Lifenet, commoning, and well-tuned provisioning. Furthermore, heterodox modeling needs to learn from existing approaches such as companion modeling or ComMod, developed by French ecologist and hydrologist Olivier Barreteau in collaboration with agronomists and human geographers. As an approach already in practice, tested and verified with a proven set of categories and roles such as lay person, researcher, technician, or student, but which wants to be both explainable and debatable at the same time, and also aiming at collective reflection, it seems highly promising to form an inspirational basis for heterodox modeling. Most notable companion-modeling approaches also include and mobilize a whole network of non-human agents into their modeling process (Barreteau et al 2014: 14–16). How ComMod could form a basis for heterodox modeling needs to be elaborated in further works.

Thirdly, even the concept of provisioning deserves reconsideration and broadening. Heterodox economics already embraced life, evolution, or the environment, but realms of provisioning at smaller scales, such as those observable in mammal physiology and cognition, are missing. Extending the notion of society towards ecosystem, as the term socio-ecological actually implies, could mean to inquire into sensorial, perceptive, affective, and cognitive ecologies of provisioning, where for example, in the most basic terms, the neuroscience of cognition in mammalian bodies explains how prediction, preparation, and prefiguration are short-circuited with action and command for a provisioning of basic bodily functions. Would a sort of Marxist-critical bionics approach offer alternative insights for social provisioning? How could we learn from how living beings, systems, and networks – and the associated evolutionary transformations – have worked out sustainable ways of provisioning?

Finally, a last, speculative proposal and “fantasy” (Foley 2020: 325) for heterodox modeling inspired by a project pursued by German commoning activist Marcus Meindel and collaborators.^12^ Meindel builds on the seminal work on commoning by Silke Helfrich (Bollier and Helfrich 2015) and proposes a patterning system to describe bottom-up provisioning processes and along the flow of materials resources and work processes. It is another proposal similar to heterodox modeling, but focuses on the fulfillment of demands and needs as described by Simon Suterlütti and Stefan Meretz (see 2023). The case-specific provisioning of basic needs such as food and nutrition is in this framework a matter to describe in a sort of modeling language, which seems to be inspired by the so-called Unified Modeling Language (UML), which is a general-purpose modeling language intended to provide software engineering standards to visualize the design of a system. These case-specific process-resource chains are fed into a database and network, linking food producers, kitchens, kitchen workers, and material flows and other necessities together into a ramified, intricate network, system, or framework probably very similar to what Foley imagined with Lifenet. Meindel’s approach tackles the question of well-tuned provisioning and commoning from the perspective of small scale bottom-up actions and processes. Combined and extended with the ideas and approaches proposed in this article, it could, similarly to the historical case with business simulation games, lead to a massively-used programming environment for heterodox modeling, which would not only propose a crucial element for the training of a new class of commonist mediators, messengers, and managers, but ultimately provide the basis for new schools of commoning and finally even convert mainstream economists, conservatives, patriarchists, fascists, liberals, capitalists, or ableists into commonists! Let us begin to fulfill our wishes and work towards making them become more real.

## Footnotes

1 In 2004 it was renamed the David A. Tepper School of Business after an American billionaire hedge fund manager, who had been a student of the same school in the early 1980s.

2 Wargaming was developed by the Prussian military as a tool for training their officers. It conventionally uses a sort of map of territories, figurines, and a set of rules. The history of wargames has been tackled by many scholars. From the perspective of media studies, the most pertinent is the work of Philipp von Hilgers, German media studies scholar and former student of Friedrich Kittler (see von Hilgers 2012).

3 A brief, but critical reading of the handbook for the first business game *Top Management Decision Simulation* (1957) will reveal this. Terms such as attack or exploit are used to describe business operations.

4 The role of scaling has been addressed by Rozas et al. (2021), proposing blockchain technology as offering possible solutions. Blockchain seems to be promising, but again it seems unrelated to the question of how the fulfillment of demands and the needs of commoners will be networked with the availability and production of commonly-shared resources. The field of blockchain is also rarely discussed in relation to modeling and simulation.

5 Surely object-oriented programming mostly led to greater profit-making and the accelerated commodification of software, but this is another story.

6 Meanwhile another programming environment and language called NetLogo emerged, which is currently more popular than StarLogo.

7 Some of them result in quite advanced choreographic performances. One such model has the unimaginative title “Cowards vs. Heroes,” and another would be Thomas Schelling’s model of segregation.

8 I found David Laibman’s work via sociologist and critical anthropologist Max Grünberg’s argumentation for “model pluralism” (Grünberg 2023: 26).

9 For this whole section on Ostrom’s game-theory-inspired arguments for common-pool resource sharing, see Ostrom 1990: 2–18.

10 For a pertinent introduction to peer production, which is a variant of commoning, see O’Neil et al. 2021. Peer production is often associated with free software culture. Historically, Yochai Benkler linked software culture and production with commons-based production quite early on, see Benkler 2002. His article notably inspired a whole new field of commoning in digital and software cultures, but the basic issues of market-driven, profit-oriented, mainstream neoclassical economics is a more profound, materialistic, and general problem: programmers and coders need food, housing, and social provisioning.

11 For a German-speaking contribution, see Gerdes et al 2021. The discussion over how modeling and gaming interrelate was one I had and then unlearned many times with Viktor Bedö, Selena Savić, and my doctoral students Yann Patrick Martins and Lisa Marie Bador.

12 Unfortunately there are no academic publications by Marcus Meindel, but there are many online resources accessible, see https://marcus-meindel.de/ or https://commoningsystem.org/.
